# Workgroup Report: Public Health Strategies for Reducing Aflatoxin Exposure in Developing Countries

**DOI:** 10.1289/ehp.9302

**Published:** 2006-08-24

**Authors:** Heather Strosnider, Eduardo Azziz-Baumgartner, Marianne Banziger, Ramesh V. Bhat, Robert Breiman, Marie-Noel Brune, Kevin DeCock, Abby Dilley, John Groopman, Kerstin Hell, Sara H. Henry, Daniel Jeffers, Curtis Jolly, Pauline Jolly, Gilbert N. Kibata, Lauren Lewis, Xiumei Liu, George Luber, Leslie McCoy, Patience Mensah, Marina Miraglia, Ambrose Misore, Henry Njapau, Choon-Nam Ong, Mary T.K. Onsongo, Samuel W. Page, Douglas Park, Manish Patel, Timothy Phillips, Maya Pineiro, Jenny Pronczuk, Helen Schurz Rogers, Carol Rubin, Myrna Sabino, Arthur Schaafsma, Gordon Shephard, Joerg Stroka, Christopher Wild, Jonathan T. Williams, David Wilson

**Affiliations:** 1 National Center for Environmental Health, Centers for Disease Control and Prevention, Atlanta, Georgia, USA; 2 International Maize and Wheat Improvement Center, Nairobi, Kenya; 3 Centre for Science Society and Culture, Indian Council of Medical Research, Hyderabad, India; 4 Kenya Medical Research Institute, Centers for Disease Control and Prevention, Nairobi, Kenya; 5 World Health Organization, Geneva, Switzerland; 6 Centers for Disease Control and Prevention, Kenya Office, Nairobi, Kenya; 7 Resolve, Washington, DC, USA; 8 Johns Hopkins Bloomberg School of Public Health, Baltimore, Maryland, USA; 9 Biological Control Center for Africa, International Institute of Tropical Agriculture, Cotonou, Benin; 10 Center for Food Safety and Applied Nutrition, U.S. Food and Drug Administration, College Park, Maryland, USA; 11 International Maize and Wheat Improvement Center, Mexico City, Mexico; 12 Department of Agricultural Economics and Rural Sociology, Auburn University, Auburn, Alabama, USA; 13 School of Public Health, University of Alabama at Birmingham, Birmingham, Alabama, USA; 14 Kenya Agricultural Research Institute, Nairobi, Kenya; 15 Institute of Nutrition and Food Safety, Chinese Center for Disease Control and Prevention, Beijing, China; 16 World Health Organization, Regional Office for Africa, Brazzaville, Republic of Congo; 17 Center for Food Risk Assessment and Quality, Istituto Superiore di Sanità, Rome, Italy; 18 Preventive and Promotive Health, Kenya Ministry of Health, Nairobi, Kenya; 19 Department of Community, Occupational, and Family Medicine, National University of Singapore, Singapore; 20 Foreign Agricultural Service, U.S. Department of Agriculture, Nairobi, Kenya; 21 Center for Food Safety, Texas A&M University, College Station, Texas, USA; 22 Food Quality and Standards Service, Food and Agriculture Organization, Rome, Italy; 23 Instituto Adolfo Lutz, São Paulo, Brazil; 24 Department of Plant Agriculture, University of Guelph at Ridgetown College, Ridgetown, Ontario, Canada; 25 Programme on Mycotoxins and Experimental Carcinogenesis, South African Medical Research Council, Tygerberg, South Africa; 26 Institute for Reference Materials and Measurements, European Commission—Joint Research Centre, Retieseweg, Geel, Belgium; 27 Molecular Epidemiology Unit, School of Medicine, University of Leeds, Leeds, United Kingdom; 28 Peanut Collaborative Research Support Program, University of Georgia, Griffin, Georgia, USA; 29 Coastal Plain Experiment Station, Department of Plant Pathology, University of Georgia, Tifton, Georgia, USA

**Keywords:** aflatoxins, biomonitoring, developing countries, food safety, hepatitis, hepatocellular carcinoma, public health, surveillance

## Abstract

Consecutive outbreaks of acute aflatoxicosis in Kenya in 2004 and 2005 caused > 150 deaths. In response, the Centers for Disease Control and Prevention and the World Health Organization convened a workgroup of international experts and health officials in Geneva, Switzerland, in July 2005. After discussions concerning what is known about aflatoxins, the workgroup identified gaps in current knowledge about acute and chronic human health effects of aflatoxins, surveillance and food monitoring, analytic methods, and the efficacy of intervention strategies. The workgroup also identified public health strategies that could be integrated with current agricultural approaches to resolve gaps in current knowledge and ultimately reduce morbidity and mortality associated with the consumption of aflatoxin-contaminated food in the developing world. Four issues that warrant immediate attention were identified: *a*) quantify the human health impacts and the burden of disease due to aflatoxin exposure; *b*) compile an inventory, evaluate the efficacy, and disseminate results of ongoing intervention strategies; *c*) develop and augment the disease surveillance, food monitoring, laboratory, and public health response capacity of affected regions; and *d*) develop a response protocol that can be used in the event of an outbreak of acute aflatoxicosis. This report expands on the workgroup’s discussions concerning aflatoxin in developing countries and summarizes the findings.

Aflatoxins, toxic metabolites of *Aspergillus flavus* and *Aspergillus parasiticus* fungi, are naturally occurring contaminants of food. Although aflatoxins have been a problem throughout history, they have been recognized as significant contaminants within agriculture only since the 1960s. The establishment of regulatory limits on traded foods, the enforcement of these limits through food monitoring, and the implementation of optimal drying and storage practices have mostly eliminated harmful exposures in developed countries (Brown et al. 1999; Phillips et al. 1994). The application of these strategies in developing countries is difficult because of differences in food production, such as the prominence of subsistence farming in developing countries. Furthermore, these countries often lack the resources, technology, and infrastructure necessary for routine food monitoring as well as optimal drying and storage practices.

Consequently, > 5 billion people in developing countries worldwide are at risk of chronic exposure to aflatoxins through contaminated foods (Shephard 2003; Williams et al. 2004). Aflatoxin-associated health effects pervade the developing world. These effects could be mitigated or prevented through effective and integrated use of current agricultural knowledge and public health practice. The discussion of this problem and its remedies must include the underlying question of food insufficiency and more general economic challenges in developing countries.

Outbreaks of acute aflatoxin poisoning are a recurrent public health problem. In 2004, one of the largest, most severe aflatoxicosis outbreaks occurred in Kenya, followed by another outbreak in 2005 [Centers for Disease Control and Prevention (CDC) 2004, unpublished data]. Both outbreaks were caused by contamination of inadequately stored, homegrown maize. Given that diseases in the developing world often go unreported, the Kenya outbreaks probably represent only a portion of the problem. The full burden of disease attributable to chronic aflatoxin exposure [e.g., hepatocellular carcinoma (HCC), impaired growth, immune suppression] remains undefined. These outbreaks emphasize the need to quantify and control aflatoxin exposure in developing countries and highlight the potential role of public health services.

In July 2005, the CDC and the World Health Organization (WHO) convened a workgroup of experts to identify culturally appropriate, long-term public health strategies to reduce aflatoxin exposure in developing countries. The 40 members included internationally recognized scientists from diverse backgrounds (public health, agriculture, animal health, trade, and social science). They also included key public health officials and stakeholders from countries heavily affected by aflatoxins. The workgroup members identified gaps in current knowledge about the acute and chronic human health effects of aflatoxins. They also reviewed surveillance and food monitoring schemes, analytic methods, and the efficacy of intervention strategies. Members discussed public health strategies that could supplement agricultural efforts to reduce or prevent exposure to aflatoxins in the developing world. Last, the workgroup discussed areas where efforts should be concentrated to reduce aflatoxin exposure and subsequently fill gaps in current knowledge.

## Background

Aflatoxins are toxic secondary metabolites produced by *Aspergillus* fungi. Aflatoxin B_1_ (AFB_1_), a known human carcinogen, is the most potent and potentially lethal metabolite. Agriculture scientists have been studying aflatoxins for > 40 years because of the widespread occurrence of those contaminants and their significant effect on crops (Eaton and Groopman 1994; Fung and Clark 2004; Shephard 2003; Wild and Turner 2002; Williams et al. 2004).

Aflatoxins can affect a wide range of commodities, including cereals, oilseeds, spices, tree nuts, milk, meat, and dried fruit. Maize and groundnuts are major sources of human exposure because of their greater susceptibility to contamination and frequent consumption throughout the world. Aflatoxins are most prevalent in latitudes between 40° N and 40° S of the equator, but the greatest health risk lies within developing countries in tropical regions, which rely on these commodities as their staple food source. Food insufficiency and lack of diversity substantially contribute to the susceptibility of individuals and communities to aflatoxins.

Contamination is influenced by many factors and can occur at any stage of food production, from preharvest to storage (Wilson and Payne 1994). Factors that affect aflatoxin contamination include the climate of the region, the genotype of the crop planted, soil type, minimum and maximum daily temperatures, and daily net evaporation (Bankole and Mabekoje 2004; Brown et al. 2001; Fandohan et al. 2005a; Ono et al. 1999; Wilson and Payne 1994). Aflatoxin contamination is also promoted by stress or damage to the crop due to drought before harvest, insect activity, poor timing of harvest, heavy rains at and after harvest, and inadequate drying of the crop before storage (Hawkins et al. 2005; Hell et al. 2000; Ono et al. 2002; Turner et al. 2005). Levels of humidity, temperature, and aeration during drying and storage are also important factors.

Acute exposure to aflatoxins can result in aflatoxicosis, which manifests as severe, acute hepatotoxicity with a case fatality rate of approximately 25% (Cullen and Newberne 1994). Early symptoms of hepatotoxicity from aflatoxicosis can include anorexia, malaise, and low-grade fever. Acute high-level exposure can progress to potentially lethal hepatitis with vomiting, abdominal pain, jaundice, fulminant hepatic failure, and death. Outbreaks of acute aflatoxicosis are a recurring public health problem in many developing countries including Kenya and India. (CDC 2004; Krishnamachari et al. 1975a, 1975b; Lye et al. 1995; Ngindu et al. 1982).

HCC as a result of chronic aflatoxin exposure has been well documented, generally in association with hepatitis B virus (HBV) or other risk factors (Chen et al. 2001; Henry et al. 2002; Omer et al. 2004; Qian et al. 1994; Wang et al. 1996). The International Agency for Research on Cancer (IARC) first recognized aflatoxins as carcinogenic in 1976. It subsequently reaffirmed naturally occurring mixtures of aflatoxins and AFB_1_ as Group 1 carcinogens (carcinogenic to humans) (IARC 2002). Additional effects of chronic exposure have not been widely studied, but are thought to include immunologic suppression, impaired growth, and nutritional interference (Cullen and Newberne 1994; Fung and Clark 2004; Patten 1981; Williams et al. 2004).

## Aflatoxins in Developing Countries

### Baseline levels of exposure

Although a few studies have provided estimates of daily exposure to aflatoxins during non-outbreak periods (Jiang et al. 2005; Park et al. 2004; Wang et al. 2001; Wild et al. 1992), more information is needed concerning baseline levels of chronic exposure for vulnerable populations. This would allow for a better understanding and quantification of the health effects associated with chronic exposure and for a better estimate of the level of aflatoxin exposure necessary to trigger an outbreak. Such knowledge would also allow for the evaluation of the efficacy of public health and agricultural interventions.

### Health impact and burden of disease caused by chronic exposure

HCC is the sixth most prevalent cancer worldwide. Developing countries have a higher incidence rate, with approximately 82% of the 600,000 new cases each year occurring in developing countries (Parkin et al. 2005). The age adjusted incidence per 100,000 in middle Africa is 27.8 for men and 13.4 for women compared with 6.2 and 1.7 in Western Europe and 5.3 and 1.9 in North America. Only China has a higher incidence, at 37.9 and 14.2.

The burden of HCC attributable to aflatoxins when accounting for comorbidities, such as HBV, is not known. Several studies in China have indicated that combined exposure to HBV and aflatoxins is associated with a much higher risk of HCC (Qian et al. 1994; Wang et al. 1996). This interaction has not been studied in other high risk areas, such as sub-Saharan Africa. The molecular mechanism of the interaction between HBV and aflatoxins also is not known (Turner et al. 2002; Wild and Turner 2002). Quantifying the proportion of HCC attributable to aflatoxin exposure, to HBV, and to the interaction of aflatoxin exposure and HBV will help identify the best public health strategies to reduce HCC, including the benefits and limits of widespread HBV vaccination.

Additional health effects associated with chronic aflatoxin exposure have not been well studied. Preliminary evidence suggests an interaction between chronic aflatoxin exposure and malnutrition, immunosuppression, impaired growth, and consequently, susceptibility to infectious diseases such as malaria and HIV/AIDS. Experimental animal evidence suggests that chronic exposure to aflatoxins may lead to impaired immunity, reduced uptake of nutrients from the diet, and growth retardation (Hall and Wild 1994; Miller and Wilson 1994). Several studies of children in Benin and Togo using aflatoxin albumin adducts as biomarkers have shown an association between aflatoxin exposure and impaired growth (Gong et al. 2002, 2003, 2004). In a recent study in Ghana, higher levels of AFB_1_–albumin adducts in plasma were associated with lower percentages of certain leuko-cyte immunophenotypes (Jiang et al. 2005). A study in Gambian children found an association between serum aflatoxin–albumin levels and reduced secretory immunoglobulin A levels in saliva (Turner et al. 2003). Although these studies show an association between aflatoxin levels and indicators of the immune system, further investigations of the impact of this association on health is needed.

The health impact of aflatoxins is complicated by exposure to multiple mycotoxins. Foods affected by aflatoxins are also susceptible to other types of mycotoxins, and multiple mycotoxins can coexist in the same commodity (Bankole and Mabekoje 2004; Fung and Clark 2004; Speijers and Speijers 2004). Therefore, individuals may be exposed to various combinations of mycotoxins (Council for Agricultural Science and Technology 2003). The health effects associated with exposure to multiple mycotoxins are not well documented (Speijers and Speijers 2004). A better understanding of exposure to multiple mycotoxins and the health effects associated with the interactions between mycotoxins would clarify the true health consequences of mycotoxins.

### Efficacy of interventions

The appropriate adaptation of commercial practices from developed countries into interventions for developing countries and information regarding the efficacy of these interventions is essential and currently missing. It is unclear whether these are applicable in developing countries because of limited resources, technology, and infrastructure as well as inherent differences in food production. For example, in Kenya, subsistence farmers consume their own grain, but they also sell part of their harvest to local markets. They may later themselves purchase grain from these markets when their own supplies are depleted (Lewis et al. 2005).

Interventions to reduce exposure to aflatoxins can occur at various stages of food production and preparation (Table 1). Interventions vary in their cost, labor intensiveness, applicability, and effectiveness in preventing aflatoxin development. The appropriate intervention or combination of interventions depends on the crop and the country. Therefore, further evaluation is needed with consideration towards the sustainability, cultural acceptability, economic feasibility, ethical implication, and overall effectiveness of potential interventions.

#### Preharvest interventions

The presence and growth of *Aspergillus* on preharvested crops can be reduced through agricultural practices such as proper irrigation and pest management. Preharvest interventions include choosing crops with resistance to drought, disease, and pests and choosing varieties that are genetically more resistant to the growth of the fungus and the production of aflatoxins (Chen et al. 2001; Cleveland et al. 2003; Cotty and Bhatnagar 1994). Elimination of inoculum sources, such as infected debris from the previous harvest, may prevent infection of the crop (Olanya et al. 1997). A biopesticide consisting of a nonaflatoxigenic strain of *Aspergillus* may competitively exclude toxic strains from infecting the crop (Cleveland et al. 2003; Dorner et al. 1999). However, the allergenic and human health aspects of the atoxigenic strain need to be evaluated.

#### Postharvest drying and storage

Before storage, properly drying crops can prevent the development of aflatoxins. Sorting and disposing of visibly moldy or damaged kernels before storage is an effective method for reducing but not eliminating the development of aflatoxins (Fandohan et al. 2005a; Turner et al. 2005). Moisture, insect, and rodent control during storage can prevent damage to the crop, which would promote aflatoxin development. Aflatoxin contamination of maize is influenced by the structure used for storage, the length of time in storage, and the form of maize stored (i.e., with husk, without husk, or loose grain) (Hell et al. 2000). A community-based intervention trial in Guinea, West Africa, focused on thorough drying and proper storage of groundnuts in subsistence farm villages (Turner et al. 2005). The trial achieved a 60% reduction in mean serum aflatoxin–albumin levels in people in intervention villages. This study illustrates that simple and inexpensive postharvest methods can have a significant impact.

#### Postharvest food preparation

Interventions during food preparation or consumption involve removing contaminated portions of food, diluting contaminated food with uncontaminated food, neutralizing aflatoxins present in food, or altering the bioavailability of the aflatoxins consumed. Simple food preparation methods such as sorting, washing, crushing, and dehulling may reduce aflatoxin levels (Fandohan et al. 2005b; Lopez-Garcia and Park 1998; Park 2002). Aflatoxins are not largely affected by routine cooking temperatures, but traditional methods of cooking food with alkaline compounds (i.e., nixtamalization) have been used to reduce aflatoxin exposure. Although the chemical reaction may temporarily inactivate aflatoxins, the reaction may then reverse in the gastric acid of the stomach (Elias-Orozco et al. 2002; Fandohan et al. 2005b; Mendez-Albores et al. 2004; Price and Jorgensen 1985).

Additional strategies for reducing aflatoxins, including enterosorption and chemoprotection, attempt to reduce the effects of aflatoxin exposure or the bioavailable portion of aflatoxins in food. Enterosorption is the use of clay, such as NovaSil, a processed calcium montmorillonite clay with a high affinity for aflatoxins (Phillips 1999; Phillips et al. 2002; Wang et al. 2005). Clay has been used as an anticaking additive in animal feed and has been shown to protect animals from ingested aflatoxins. Chemoprotection is the use of chemical {e.g., oltipraz [4-methyl-5-(2-pyrazinyl)-1,2-dithiole-3-thione], chlorophylin} or dietary intervention (e.g., eating broccoli sprouts, drinking green tea) to alter the susceptibility of humans to carcinogens and has been considered as a strategy to reduce the risk of HCC in populations with high exposures to aflatoxins (Bolton et al. 1993; Kensler et al. 1994, 2004; Wang et al. 1999). These strategies, however, are expensive and are therefore difficult to implement in poor communities. The efficacy, safety, and acceptability of enterosorption and chemoprotection require further study.

#### Awareness campaigns

During the 2005 Kenya outbreak, individuals who received information on maize drying and storage through an awareness campaign run by the Food and Agricultural Organization and Kenya’s Ministry of Health and Ministry of Agriculture had lower serum aflatoxin levels than those who did not receive this information (CDC, unpublished data). Awareness campaigns should use systems that are in place already for disseminating information to subsistence farmers (James 2005). Awareness campaigns should distribute information to multiple organizations and use multiple means for spreading information to reach a broad range of people, given the diversity of cultures and remoteness of villages. Organizations providing information need to identify groups that are not receiving messages from current campaigns and appropriate methods for reaching those populations. They should also determine why individuals or groups are unwilling or fail to adopt recommendations.

### Analysis of food and biologic specimens

Determining the relationship between aflatoxin concentrations in food or biologic specimens and potential health outcomes is central to quantifying and mitigating the aflatoxin burden in the developing world. To improve public health, the goals of toxicologic laboratory testing include

- establishing a baseline in humans and the environment (e.g., foods, communities, individuals)
- monitoring exposure
- confirming exposure or diagnosis of poisoning
- excluding other causes of disease
- monitoring the effectiveness of prevention interventions
- and guiding therapeutic interventions.

Interpretation and application of aflatoxin results to achieve these goals are limited and vary with the type of laboratory method and sample media.

#### Aflatoxin food concentrations

Testing food for aflatoxins is constrained by two limitations. First, obtaining a representative sample of food from subsistence farmers is difficult given the need for large samples, multiple vulnerable crops on one farm, the distance between farmers, villages, and laboratories, and uneven distribution of aflatoxin contamination within a food supply.

Second, little is known about the specific threshold levels associated with adverse health effects. Agricultural data have established a relationship between concentrations of aflatoxins in food and acute aflatoxicosis. This has led to regulatory limits on aflatoxin in feed of 100–300 ppb for mature animals and 20–100 ppb for immature and dairy animals in the United States (Phillips et al. 1994). Limits for foods for human consumption in the industrialized world (including exports from developing countries) are 4–20 ppb; those limits are based on limited information from risk assessments of HCC (Henry et al. 1999; van Egmond 2002). Information is extremely limited concerning health effects associated with aflatoxin concentrations between 20 ppb and 300 ppb.

#### AFB_1_ adducts and urine immunoassay

For epidemiologic studies, biomarkers in serum and urine provide a better estimate of aflatoxin exposure than does food analysis. Aflatoxin metabolites in urine reflect recent exposure (i.e., 2–3 days), whereas aflatoxin albumin adducts in blood reflect exposure over a longer period (i.e., 2–3 months) (Groopman et al. 1994). These analyses, however, are labor intensive and expensive (McCoy et al. 2005; Sheabar et al. 1993; Wild et al. 1990).

Information regarding the interpretation and application of AFB_1_ adducts and urine immunoassays is also limited (Groopman and Kensler 2005; Turner et al. 1998; Wild and Turner 2001). Aflatoxin metabolites or adducts in urine and serum indicate exposure, but do not necessarily equate to adverse health effects. Some studies have examined the correlation of aflatoxin intakes to biomarker levels (Groopman et al. 1992; Wild et al. 1992) and to disease (Azziz-Baumgartner et al. 2005; Gong et al. 2004; Qian et al. 1994; Wang et al. 1996). More research is needed to further elucidate the correlation between aflatoxin levels in biologic specimens and adverse health effects. Research must also clarify the relationship between aflatoxin levels in biologic specimens and levels in food.

### Appropriate laboratory methods for developing countries

Current methods can detect very low levels of aflatoxins and aflatoxin metabolites in food and biologic media. The use of these methods within developing countries is limited by practical considerations, such as resources and infrastructure. Methods for testing food and biologic specimens need to be adapted to fit the surveillance and epidemiologic needs of developing countries. A simple screening method, adapted for developing countries, would benefit subsistence farmers and be useful to public health and agriculture institutions. These institutions would also benefit from sustainable and reliable confirmatory methods for use in centralized laboratories.

#### Field methods

Simple and inexpensive field screening methods, such as portable, lateral flow immunochromatographic assays, are available to determine that food is sufficiently free of aflatoxins. Field methods can be performed with minimal training or equipment and can be performed onsite (i.e., at a farm or grain silo). Field methods for aflatoxin analysis allow for rapid confirmation or exclusion of possible exposure at a reasonable cost, thus allowing officials to quickly determine the need for further evaluation and intervention. Such methods would prove beneficial in developing countries where the remoteness of villages and long distances to a centralized laboratory make it impractical to take samples from villages, analyze them in the laboratory, and then travel back to the village to deliver the results.

Currently, however, these lack direct applicability in developing countries. For example, one field screening method uses dipsticks that indicate whether a sample is above or below the regulatory limit of 20 ppb. In developing countries, especially during an outbreak, most samples would be > 20 ppb. Therefore, an investigator would need to differentiate between samples at levels > 20 ppb. Such field tests could prove effective if they were adjusted to action levels suitable for developing countries. Field methods for the analysis of biologic samples have not been developed. However, the same concept of using dipsticks can be applied to field tests for biologic specimens. Efforts to limit aflatoxin exposure in developing countries could be enhanced by reducing the cost and improving the durability, ease of transport, and usability of field methods. Ideally, such methods should be easy to use and should not require electricity.

#### Laboratory methods

Laboratory methods can be used to confirm results of field tests. They are more precise, but also more labor intensive and costly. These methods require instrumentation or techniques not suited to working onsite. They require regular maintenance of instrumentation, training of personnel, and a ready supply of reagents and materials (Trucksess and Wood 1994). The best laboratory method for testing either food or biologic specimen is one that balances the need for quick, accurate results with limitations in resources and infrastructure. Current laboratory methods require further refinement to improve their usability in developing countries. Thin-layer chromatography is a well-suited laboratory method for testing food samples, given its reliability and simplicity (Shephard and Sewram 2004; Stroka and Anklam 2000). It is labor intensive, however, and limited in the number of samples that can be tested in a day. Alternatives for food analysis include commercially available aflatoxin testing kits, which are less labor intensive and faster, but also more expensive (Scott and Trucksess 1997).

### Early warning system for developing countries

**Figure 1:**
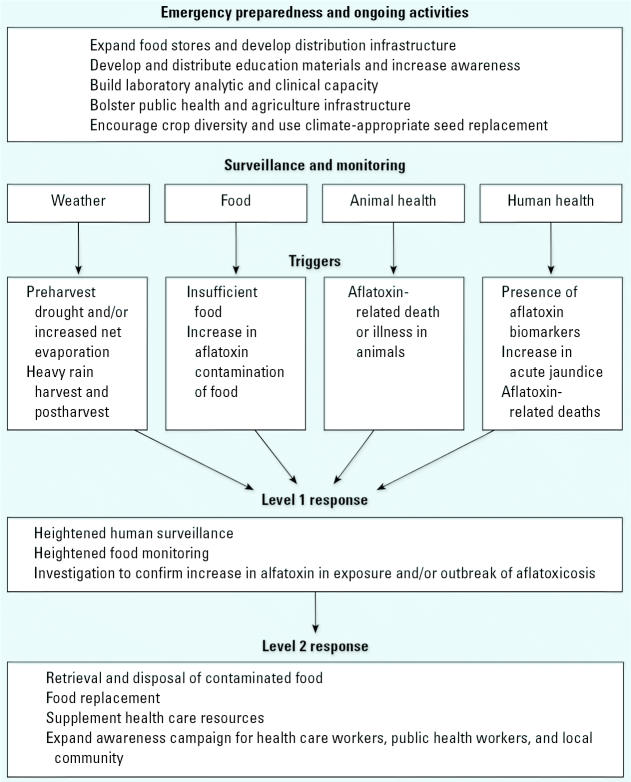
Overview of preparedness, surveillance, and response activities for preventing acute aflatoxico-sis in countries in development.

To prevent future outbreaks, developing countries could benefit from an early warning system designed to detect food contamination that could cause illness (Figure 1) (Park 1995). Public health surveillance is the ongoing systematic collection, analysis, interpretation, and dissemination of data regarding a health-related event. Those data are used in public health actions to reduce morbidity and mortality and to improve health (CDC 2001). To create an effective and sustainable system, health surveillance and food and biologic monitoring strategies must be adapted to meet the needs of developing countries. Early warning signs need to be validated and response protocols need to be developed.

Previous outbreaks in Kenya have been identified by physicians who noticed an increase in cases of jaundice, despite a lack of any organized or official reporting system (Azziz-Baumgartner et al. 2005). Although a national reporting system for jaundice would prove beneficial for developing countries, the baseline rate of jaundice and all its possible causes are not known. In addition, aflatoxicosis confirmation tests using biologic markers are limited. However, an active and organized reporting system of possible aflatoxin cases may allow for earlier detection of potential outbreaks.

An early warning system should also involve monitoring aflatoxin levels in food sources or individuals to prevent or reduce the health affect. Monitoring aflatoxin levels in food or individuals to identify those at risk for disease is more difficult than monitoring rates of jaundice. However, food and biologic monitoring may identify susceptibility sooner and allow for a more timely intervention. A robust monitoring or surveillance system would be difficult to establish and sustain. To maximize resources, a targeted monitoring or surveillance system for high-risk areas or populations should be used. The specimen (food, urine, or serum) most appropriate for the country’s capacity should be collected. A combination of rapid field test and laboratory confirmation tests that analyzes aflatoxins in food or biologic samples would be ideal for an early warning system.

In addition to increase in jaundice cases or the detection of aflatoxin in food and biologic samples, other factors that indicate or influence aflatoxin contamination could be incorporated. Deaths of livestock or domestic animals, which are often given lower-quality grain, or modeling of aflatoxin contamination to weather conditions from planting to postharvest (de la Campa et al. 2005), could also serve as indicators of aflatoxin. Both would require further validation and an infrastructure for monitoring and dissemination of information. Ultimately, an early warning system should rely on multiple sources of information and triggers that would set in motion various responses for preventing or reducing an outbreak of aflatoxicosis.

An early warning system must include a response protocol to prevent further aflatoxin exposure and associated health outcomes once a contaminated food source is identified. A protocol can be effective only if the infrastructure and funds to replace contaminated food exist and a method for identifying families in need has been determined. For an early warning system to succeed, key members from various government agencies, the health care sector, and nongovernmental organizations need to be part the development and implementation of effective communication and response strategies.

## Conclusions

The aflatoxin workgroup brought together a diverse group of experts to identify public health strategies, which complement agricultural strategies, to reduce aflatoxin exposure in developing countries. Although a great deal is known about aflatoxins, little is known about aflatoxin exposure and the resulting health effects in developing countries. Even without a complete understanding of the public health problem caused by aflatoxins, it is clear that acute aflatoxicosis is preventable and chronic exposure can be reduced. Efforts to reduce aflatoxin exposure require the commitment of sufficient resources and the collaboration between the agriculture and public health communities and between the local, regional, national, and international governing bodies. Four issues that warrant immediate attention include:

- quantifying the human health impacts and the burden of disease due to aflatoxin exposure
- compiling an inventory, evaluating the efficacy, and disseminating results of ongoing intervention strategies
- developing and augmenting the disease surveillance, food monitoring, laboratory, and public health response capacity of affected regions
- and developing a response protocol that can be used in the event of an outbreak of acute aflatoxicosis.

These steps will provide much needed knowledge about the pattern and resulting health effects of aflatoxin exposure and will enable the development of effective, culturally appropriate interventions for reducing chronic levels of exposure. Although aflatoxin exposure is not a new issue, it requires new strategies to address it effectively within developing countries, where aflatoxin exposure is intertwined with the issues of food insecurity and insufficiency. The consecutive outbreaks in Kenya emphasize the imperative for action.

## Figures and Tables

**Table 1:** Interventions for preventing or reducing aflatoxin exposure.

Stage in food production	Interventions	References
Preharvest	Timing of planting; crop planted; genotype of seed planted; irrigation; insecticides; competitive exclusion; timing of harvest	Brown et al. 2001; Chen et al. 2001; Cleveland et al. 2003; Cotty and Bhatnagar 1994; Dorner et al. 1999; Munkvold 2003; Wilson and Payne 1994
Postharvest: drying and storage	Hand sorting; drying on mats; sun drying; storing bags on wooden pallets or elevated off ground; insecticides; rodent control	Fandohan et al. 2005a; Hawkins et al. 2005; Hell et al. 2000; Munkvold 2003; Ono et al. 2002; Turner et al. 2005
Postharvest: food preparation	Hand sorting; winnowing; washing; crushing and dehulling; nixtamalization; acidification; chemoprotectant; enterosorption	Castells et al. 2005; Elias-Orozco et al. 2002; Fandohan et al. 2005b; Kensler et al. 2004; Mendez-Albores et al. 2005; Mendez-Albores et al. 2004; Munkvold 2003; Price and Jorgensen1985; Wang et al. 2005
